# In Vivo Assessment of Laboratory-Grown Kidney Tissue Grafts

**DOI:** 10.3390/bioengineering10111261

**Published:** 2023-10-29

**Authors:** Tinghsien Chuang, Justin Bejar, Zhiwei Yue, Mary Slavinsky, Denise Marciano, Iain Drummond, Leif Oxburgh

**Affiliations:** 1 The Rogosin Institute, New York, NY 10021, USA; 2 Division of Nephrology, Department of Internal Medicine, Department of Cell Biology, University of Texas Southwestern Medical School, Dallas, TX 75390, USA; 3 Mount Desert Island Biological Laboratory, Bar Harbor, ME 04609, USA

**Keywords:** kidney organoid, subcapsular engraftment, human urine biomarkers

## Abstract

Directed differentiation of stem cells is an attractive approach to generate kidney tissue for regenerative therapies. Currently, the most informative platform to test the regenerative potential of this tissue is engraftment into kidneys of immunocompromised rodents. Stem cell-derived kidney tissue is vascularized following engraftment, but the connection between epithelial tubules that is critical for urine to pass from the graft to the host collecting system has not yet been demonstrated. We show that one significant obstacle to tubule fusion is the accumulation of fibrillar collagens at the interface between the graft and the host. As a screening strategy to identify factors that can prevent this collagen accumulation, we propose encapsulating laboratory-grown kidney tissue in fibrin hydrogels supplemented with candidate compounds such as recombinant proteins, small molecules, feeder cells, and gene therapy vectors to condition the local graft environment. We demonstrate that the AAV-DJ serotype is an efficient gene therapy vector for the subcapsular region and that it is specific for interstitial cells in this compartment. In addition to the histological evaluation of epithelial tubule fusion, we demonstrate the specificity of two urine biomarker assays that can be used to detect human-specific markers of the proximal nephron (CD59) and the distal nephron (uromodulin), and we demonstrate the deposition of human graft-derived urine into the mouse collecting system. Using the testing platform described in this report, it will be possible to systematically screen factors for their potential to promote epithelial fusion of graft and host tissue with a functional intravital read-out.

## 1. Introduction

The Centers for Disease Control and Prevention (CDC) estimates that approximately 15% of the adult population of the United States has chronic kidney disease (CKD) [1]. CKD is commonly caused by hypertension and type II diabetes and also occurs as a direct sequel to acute kidney injury (AKI) following ischemic events and sepsis. End-stage kidney disease (ESKD) occurs when function is impaired to the point that basic physiological functions cannot be met, and renal replacement therapy becomes necessary. Complete organ replacement by allogeneic transplantation of a kidney is possible for individuals who are eligible and who can obtain an organ. However, this option is available to only a minority of ESKD patients. The 2018 statistics from the United States Renal Data System show that there were 229,887 patients with a functioning kidney graft, while 554,038 patients were undergoing dialysis [2]. Compared with dialysis, long-term survival is significantly improved in patients receiving grafts [3]. Quality of life is significantly reduced for many dialysis patients, who are frequently affected by depression, sleep disturbances, and restless legs syndrome [4]. Kidney transplantation is thus preferable for many different reasons, but the availability of donor kidneys is severely limited.

One potential solution to the kidney organ shortage is the production of kidney tissue from pluripotent stem cells (PSCs). This could potentially yield a limitless supply of kidney tissue for use in regenerative medicine applications. Protocols have been described for kidney differentiation of PSCs [5,6,7], and several groups are working to improve the fidelity of the tissue to natural adult kidney tissue [8,9]. Challenges include the limited maturity of tissue and its lack of organization. Engraftment of stem cell-derived kidney tissue into adult mice promotes tissue maturity, improves structural organization [10,11], and provides a platform with which to study the functional capacity of this tissue in vivo. Stem cell-derived kidney tissue grafted under the kidney capsule of immunocompromised mice is vascularized by the host, and variable contribution of vasculature from the graft itself has been reported [11,12]. Vascularization ensures that blood is fed into the glomerulus, where size-specific sieving takes place [13], and blood filtrate is fed into the nephron tubules of the graft. However, no reports have described urine outflow from the graft to the host collecting system, and all evidence indicates that fluid accumulates in blind-ending nephron tubules within the graft. Because there is no epithelial conduit for urine produced through filtration in the graft, it does not attain its full physiological function and it degenerates within weeks of surgery. Solving the problem of connecting epithelial tubules in the graft with the tubular collecting system of the host is essential to studying the physiological functionality of stem cell derived tissue, and also to evaluating its potential for long-term maintenance of its differentiation state. 

The goal of this series of investigations was to establish a technical platform that can be used for the co-implantation of laboratory-grown kidney tissue with factors that condition the local graft environment under the capsule of the kidney. The subcapsular location for implantation was selected because it places the graft tissue in direct contact with the cortex of the kidney. Adult kidneys have a superficial network of blood vessels surrounding the tubules under the capsule from which vessel sprouts can be recruited into the graft tissue as well as superficially located collecting ducts (CDs), which are the targets for epithelial fusion as these are the conduits that lead urine to the pelvis of the kidney. Repeated engraftment studies by our laboratory have not revealed any spontaneous capacity for epithelial connection between grafted tissue and host tissue, while vascularization of the graft by the host is vigorous [10,12]. Because the end-point of blood filtration is the production of urine, the lack of contiguous epithelial tubules between the graft and the host is a major bottleneck in the advancement of regenerative therapy based on generating new kidney tissue. 

Epithelial fusion is a complex biological process that occurs in strictly defined contexts during fetal development, such as the fusion of palate shelves and the addition of new nephrons to the collecting duct system of the kidney. The paucity of literature describing similar fusion events in adults suggests that this process may be restricted to the fetal period. Numerous groups have reported outcomes of grafting kidney organoids under the capsule of the mouse kidney, but to date, no evidence of epithelial fusion has been presented. We hypothesize that it will be necessary to condition the local environment to recapitulate an embryonic environment conducive to epithelial fusion to generate urine outflow from nephrons in the graft into the host collecting system. For this purpose, we aimed to develop a grafting system in which laboratory-grown kidney tissue could be assembled together with recombinant proteins, feeder cells, or gene therapy vectors in a single construct for implantation. We also aimed to identify human-specific biomarkers that can be used to detect human-derived urine against the background of mouse urine produced by the host. 

## 2. Materials and Methods

### 2.1. Mice

*Gt(ROSA)26Sor^tm4(ACTB-tdTomato,-EGFP)Luo^*/J (mTmG), NOD.Cg-*Prkdc^scid^ Il2rg^tm1Wjl^*/SzJ (NSG) and C57BL/6J (BL6) strains purchased from The Jackson Laboratory were used in this study. Animal care was in accordance with the National Research Council Guide for the Care and Use of Laboratory Animals, and all experiments were approved by the Institutional Animal Care and Use Committee of the New York Blood Center, where the Rogosin Institute laboratory is located.

### 2.2. Preparation and Gelation of Fibrinogen

Human (MilliporeSigma, Burlington, MA, USA), bovine (MilliporeSigma, Burlington, MA, USA), and salmon (Salmonics LL, Brunswick, ME, USA) plasma fibrinogen were reconstituted according to the manufacturers’ instructions and sterility-tested by inoculation of cell culture growth medium containing DMEM (ThermoFisher Scientific, Waltham, MA, USA) and 10% fetal bovine serum (R&D Systems, Minneapolis, MN, USA). The protein concentration of the fibrinogen solution was measured by BCA assay (ThermoFisher Scientific, Waltham, MA, USA). Fibrinogen was clotted by the addition of human thrombin (MilliporeSigma, Burlington, MA, USA) at a final concentration of 20 units per mL. PBS without calcium or magnesium (ThermoFisher Scientific, Waltham, MA, USA) was used as a diluent for the preparation of clots. Clots were generated by pipetting directly onto the surface of sterile petri dishes in 8 µL droplets with as small a circumference as possible. For curing, Petri dishes were incubated in a 37 °C humidified incubator for 30 min and then transferred to ice for 30 min.

### 2.3. Culture of Kidney Organoids

Human kidney organoids were generated according to the protocol described in [7]. All pluripotent stem cell cultures were performed using Stemfit Basic 04 (AMSBIO LLC Cambridge, MA, USA), and all directed differentiation cultures were performed using Advanced RPMI 1640 (ThermoFisher Scientific, Waltham, MA, USA) supplemented with glutamine (ThermoFisher Scientific, Waltham, MA, USA)—hereafter referred to as ARPMI/G. Briefly, pluripotent stem cells were cultured on wells coated with Matrigel (Corning Inc., Corning, NY, USA) in 6-well plates. Cells were detached using TrypLE Express Enzyme solution (ThermoFisher Scientific, Waltham, MA, USA), which was neutralized by adding an equal volume of Stemfit Basic 04, then pelleted by centrifugation and replated in medium containing 10 µM Y-27632 Rho kinase inhibitor (MilliporeSigma, Burlington, MA, USA), which was replaced with medium without inhibitor after 16 h. In preparation for differentiation, 90,000 cells were seeded in one well of a 6-well plate (Corning Inc., Corning, NY, USA) coated with Matrigel and allowed to grow in Stemfit 04 Basic for 2 days. The medium was replaced with ARPMI/G containing 9 µM CHIR99021 (ReproCELL USA Inc., Prince George’s County, MD, USA), which was replenished after 48 h. After a total of 96 h treatment with CHIR99021, the medium was removed and cells were gently rinsed with PBS before the addition of ARPMI/G containing 10 ng/mL Activin A (R&D Systems, Minneapolis, MN, USA), which was replenished after 48 h. After a total of 72 h treatment with Activin A, the medium was removed and cells were gently rinsed with PBS before the addition of ARPMI/G containing 10 ng/mL human FGF9. After a total of 48 h treatment with FGF9, cells were detached from the wells using TrypLE Express enzyme solution. The enzyme was neutralized by the addition of an equal volume ARPMI/G containing 10% fetal bovine serum (R&D Systems, Minneapolis, MN, USA), strained through a 30 µM strainer (Miltenyi Biotec Inc., Auburn, CA, USA), and pelleted by centrifugation at 300× *g*. The pellet was resuspended at 500,000 cells per mL in ARPMI/G containing 10 ng/mL FGF9, and 200 µL was dispensed per well in a 96-well ultralow attachment plate (Corning Inc., Corning, NY, USA), which was centrifuged for 20 s at 300× *g*. 100 µL of the medium was replaced with ARPMI/G containing 20 ng/mL FGF9 after 2 days. Thereafter, 100 µL medium was replaced every 2 days with ARPMI/G for the duration of the culture period. Human kidney organoids were generated from WA09 (also known as H9) human embryonic stem cells (license 23-W0172 from WiCell Research Institute Inc., Madison, WI, USA). 

Mouse kidney organoids were generated according to the protocol described in [14]. Briefly, nephrogenic zone cells were enzymatically isolated from P0 kidneys of heterozygous mTmG mice using a pancreatin/collagenase cocktail. Then, the mixture was strained through a 30 µm strainer, suspended in APEL 2 medium (Stem Cell Technologies) containing 10 µM CHIR99021 at 50,000 cells per µL. Then, 2 µL droplets were spotted onto 0.1 µm pore size polycarbonate filters (MilliporeSigma, Burlington, MA, USA), which were suspended on 1 mL APEL 2 medium containing 50 ng/mL FGF9 in a 24-well plate. The medium was replaced with APEL 2 containing FGF9 after 48 h, and subsequently, the medium was replaced with APEL 2 every 48 h. 

### 2.4. Adeno Associated Virus (AAV)

Reporter AAV viruses expressing GFP under the control of the CMV promoter were purchased from Vectorbuilder Inc., Chicago, IL, USA. AAV-DJ-EF1A-dTomato was assembled and produced at greater than 1 × 10^13^ gene counts per milliliter by Vectorbuilder Inc., Chicago, IL, USA. 

### 2.5. Surgery 

NSG mice used as hosts for grafting studies were used at 8–12 weeks of age, and all experiments were performed in females. Mice were anesthetized using isoflurane (Med-Vet International, Mettawa, IL, USA), and 5 mg/kg of the analgesic Meloxicam (Covetrus, Portland, ME, USA) was administered daily for 72 h after surgery. Subcapsular implantation was performed as described in [15]. 

### 2.6. Tissue Preparation and Staining

Kidneys were harvested from euthanized mice, and the region surrounding the implant was dissected out and fixed immediately in 4% paraformaldehyde (MilliporeSigma, Burlington, MA, USA) dissolved in PBS (MilliporeSigma, Burlington, MA, USA). After 2 h fixation, tissue slabs were equilibrated to 10% sucrose (MilliporeSigma, Burlington, MA, USA), then 20% sucrose dissolved in PBS. Tissue was then cryo-embedded in Tissue-Tek O. C. T. compound (Sakura Finetek USA Inc., Torrance, CA, USA) and sectioned in a cryostat at 10 µm. Slides were immunostained as described in [10] using primary antibodies listed in Supplementary Table S1, all at 1:100 dilution. Fluorescently labeled secondary antibodies were applied at 1:200 dilution. Fluorescently labeled slides were visualized using a compound microscope.

### 2.7. Urine Samples

Mouse urine samples were harvested from 8–12-week-old male and female BL6 mice, centrifuged at 5000× *g* for 5 min to remove particles and frozen at −80 °C until use. Anonymized male and female human urine samples were purchased from BioIVT, Westbury, NY, USA, sedimented, and stored at −80 °C until use. Because these biospecimens were not associated with any information enabling identification of the donor and were purchased from a commercial vendor, their use was exempted from IRB review.

### 2.8. ELISA

Assays for uromodulin (Human Uromodulin DuoSet ELISA DY5144-05, R&D Systems, Minneapolis, MN, USA), CD59 (Human CD59 ELISA ab263893, Abcam, Waltham, MA, USA), PSAP (Human PSAP/Prosaposin (Sandwich ELISA) ELISA LS-F35235, Lifespan Biosciences, Lynnwood, MA, USA), cubilin (Human CUBN/Cubilin (Sandwich ELISA) ELISA LS-F38077, Lifespan Biosciences, Lynnwood, MA, USA), SPP1 (Human Osteopontin (OPN) Quantikine ELISA DOST00, R&D Systems, Minneapolis, MN, USA), and HSPG2 (HSPG2 elisa kit: Human Basement membrane-specific heparan sulfate proteoglycan core protein ELISA MBS765938, MyBioSource, San Diego, CA, USA) were used according to the manufacturer’s instructions and assays were read on a Glomax Explorer (Promega, Madison, WI, USA) plate reader.

## 3. Results

### 3.1. A Fibrin-Based Vehicle for Subcapsular Engraftment

Separation of the kidney capsule from the cortex of the kidney causes a small amount of bleeding due to the rupture of superficial blood vessels, leading to the formation of a blood clot around subcapsularly implanted material. This blood clot is degraded by endogenous processes and is generally not visible one week after surgery. Based on the capability of endogenous processes to clear the blood clot, we reasoned that a hydrogel based on the major clot component fibrin would be a suitable vehicle to encapsulate graft tissue. Furthermore, fibrin acts as an efficient scaffold for blood vessel in-growth [16], which is a key determinant of graft survival. 

**Figure 1:**
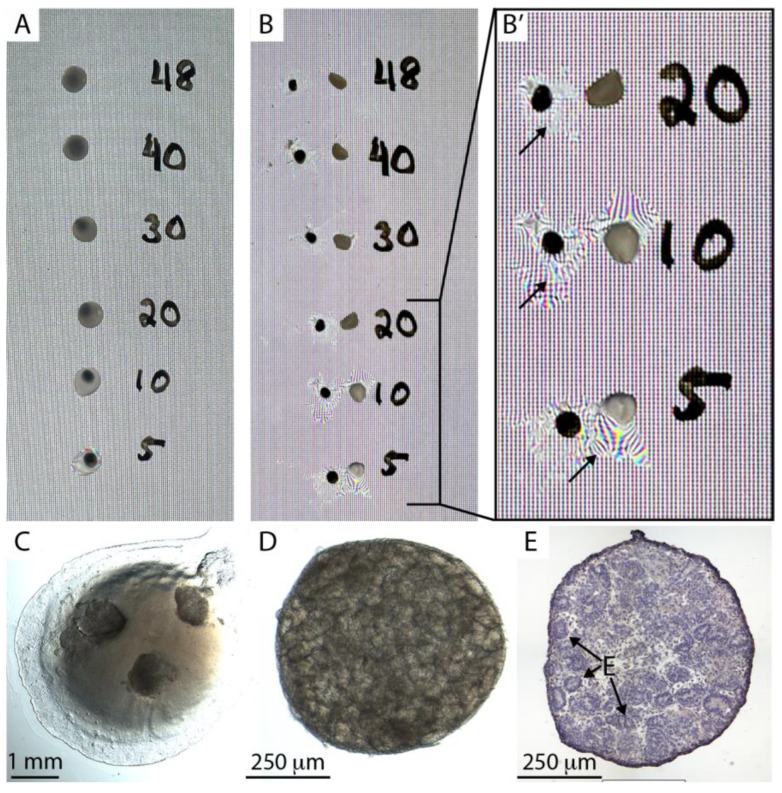
Fibrin-embedding organoids. (**A**). 8 µL spots of fibrinogen:thrombin mix were spotted on a petri dish at concentrations 5–45 mg/mL and cured for 30 min at 37 °C and then for 30 min on ice. Note that solutions of 20 mg/mL concentration or higher display strong turbidity. (**B**). Fibrin clots were dislodged from the petri dish, picked up with forceps, and placed back on the dish. (**B′**) Manual handling of clots with lower fibrinogen concentration than 30 mg/mL caused collapse of the hydrogel and loss of water content (arrows). (**C**). Three human stem cell-derived kidney organoids embedded in an 8 µL fibrin clot on the base of a Petri dish. (**D**). A human kidney organoid after overnight storage on ice. (**E**). Hematoxylin-stained cryosection of a human kidney organoid after overnight storage showing epithelia (**E**).

Salmon, bovine, and human fibrinogen were clotted at stock concentrations by the addition of 20 units/mL of human thrombin. They were all clotted to firm hydrogels within 30 min and are suitable candidates for experimental grafting. Because our objective was to develop a system for testing engraftment of human kidney tissue into immunocompromised mice, we selected human fibrinogen for further evaluation. Two features are essential for the hydrogel composition: First, the hydrogel must gelate within thirty seconds to a minute, providing sufficient working time to prepare constructs and prevent gelation in the pipette tip. Second, the fibrin hydrogel must be sufficiently rigid so that it can be picked up with forceps during surgery without collapsing. A series of fibrinogen concentrations from 5 to 48 mg/mL was prepared, mixed with 0.02 units of human thrombin, and spotted onto the surface of a Petri dish, where they were gelated for 30 min in a humidified 37 °C incubator (Figure 1A). Clots were separated from the surface of the dish using a scalpel and picked up with dissecting forceps (Figure 1B). We found that the minimal fibrinogen concentration that could form a hydrogel sufficiently rigid to be separated from the plate surface and picked up without collapsing and losing its liquid content was 30 mg/mL (Figure 1B′). We found that an additional 30 min curing time on ice increased the mechanical stability of the fibrin. The protocol used for all remaining work in this report was as follows:

1. Thaw fibrinogen and thrombin stocks on ice.
2. On ice, mix desired components (e.g., feeder cell suspension, recombinant proteins, virus), and stock fibrinogen and PBS (no Ca or Mg) to a final concentration of 33 mg/mL fibrinogen. Make 8 µL per clot and reserve on ice.
3. Pipette 0.8 µL thrombin into one Eppendorf tube for each clot to be made and place on ice.
4. Place organoids in 8 µL droplets of PBS on the surface of a Petri dish, one droplet per clot separated by at least 2 cm—it is important to make as small a footprint of PBS as possible with the droplet because it will determine the final diameter of the fibrin clot.
5. From this point on, make the clots one at a time.
6. Remove PBS from one droplet containing organoids. Make sure to drain as thoroughly as possible. Otherwise, the concentration of fibrinogen will be inaccurate.
7. Withdraw 7.2 µL fibrinogen mix using a Gilson P10 or P20, and while the liquid is still in the tip, adjust the volume of the pipette to 8 µL.
8. Depress the plunger enough to expel the air and pipette the fibrinogen solution into one Eppendorf tube containing thrombin.
9. Rapidly aspirate and expel three times to mix the components. Make sure you do not introduce air bubbles.
10. Immediately pipette the solution on top of the organoids without increasing the size of the footprint created by the PBS droplet.
11. After all clots have been pipetted, transfer them to a 37 °C humidified incubator.
12. After 30 min at 37 °C, keep on ice for 30 min.
13. For surgery, it is most convenient to flood the dish with serum-free medium and keep the plate on ice. During surgery, detach the clots from the plate immediately before implantation by cutting with a scalpel as close to the dish surface as possible and implant under the kidney capsule with the side that was attached to the dish facing the cortex. This ensures that the organoids are in direct contact with the kidney cortex.
14. Alternatively, the clots can all be detached after curing and placed in a tube or Petri dish containing a few mL of serum-free medium on ice awaiting surgery—this is most convenient if clots are to be transported, but the disadvantage is that it is difficult to distinguish the two different sides of the clot when doing the surgery.

Kidney organoids derived from H9 human embryonic stem cells [7] were embedded in a fibrin clot on the surface of a petri dish (Figure 1C). The dish was then flooded with serum-free medium, stored on ice overnight, and subsequently imaged using a stereomicroscope (Figure 1D), cryosectioned, and stained (Figure 1E). Organoids showed abundant epithelial tubules and no overt signs of cell death, such as nuclear condensation, suggesting that organoids in fibrin clots can be stored overnight for transportation. Similar observations were made with organoids generated from primary mouse nephron progenitor cells [14].

**Figure 2:**
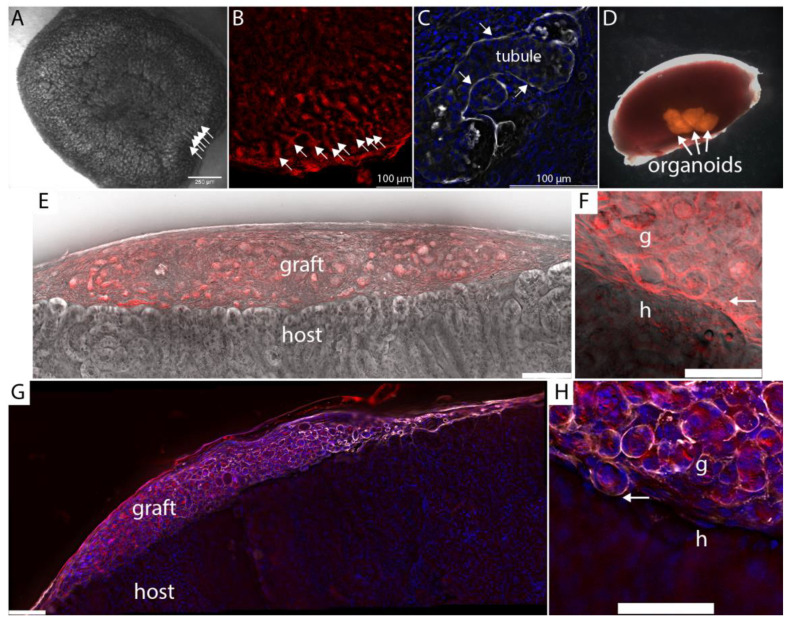
Subcapsular engraftment of kidney organoids. (**A**). Kidney organoid generated from mouse primary cells showing densely packed tubules (arrows). (**B**). Organoids were generated from the mTmG mouse strain that has ubiquitous expression of fluorescent protein associated with cell membranes. Densely packed tubules at the edge of the organoids are indicated by arrows. (**C**). Laminin immunostaining of basement membrane in cryosection of organoid shown in panel B. (**D**). Mouse kidney with 4 fibrin-embedded mTmG kidney organoids grafted under the capsule. (**E**). Host kidney cryosectioned 7 days after engraftment and visualized using autofluorescence in the Cy5 channel (grey) versus Texas red. (**F**). Higher magnification of the graft:host interface of the tissue shown in panel E. Arrow points to the boundary between graft and host. (**G**). Laminin-1 antibody staining (white) shows strong basement staining in the graft (red) but modest staining in the host tissue. (**H**). Higher magnification of laminin-1 staining at the graft: host interface (arrow).

To test the utility of fibrin clots in subcapsular implantation, we generated triplicate fibrin clots containing mouse kidney organoids from the mTmG strain, which has ubiquitous expression of cell membrane-associated Tomato fluorescent protein (Figure 2A,B). Organoids showed densely packed tubules (arrows in Figure 2A,B), which were confirmed by cryosectioning and immunostaining for the basement membrane protein laminin-1 (Figure 2C). Four mTmG organoids were embedded in each fibrin clot, and one fibrin clot per mouse was implanted under the kidney capsule of three immunocompromised (NSG) mice (Figure 2D). Because the fibrin clot is robust and can easily be handled with forceps, the implantation procedure was extremely efficient, averaging only a few minutes per mouse. Furthermore, compared with our traditional approach of implanting several organoids either one at a time into the same implantation site or by injection in a slurry, which places the tissue in an approximate location prone to movement after the surgery, retention of fibrin-embedded organoids at the implantation site was flawless.

**Figure 3:**
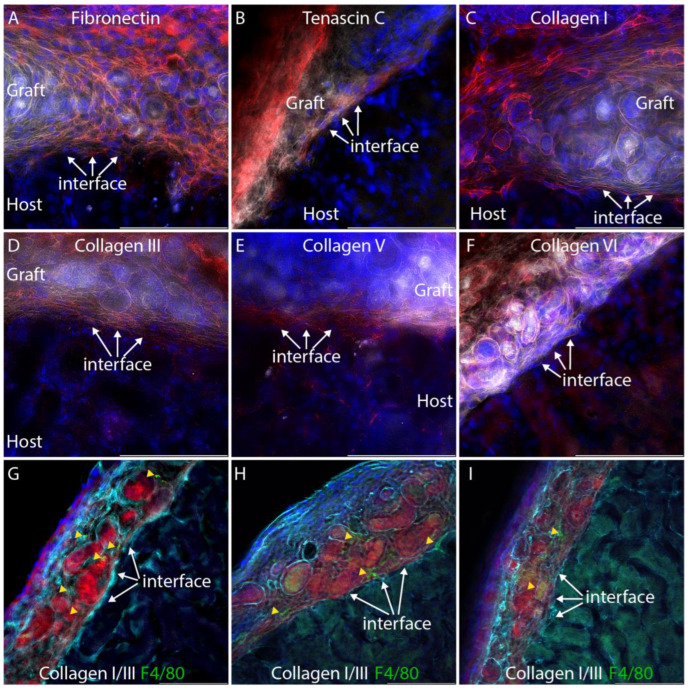
Extracellular matrix staining at the graft:host interface. For clarity, the tdTomato fluorescent signal from the graft is pseudocolored white and the extracellular matrix immunostain is pseudocolored red. DAPI nuclear stain is shown in blue. (**A**–**E**). Fibronectin, tenascin C, collagen I, collagen III, and collagen V all accumulate at the graft host interface (arrows). (**F**). Collagen VI is not seen at the graft:host interface (arrows). (**G**–**I**). Composite immunostaining for collagens I and III to visualize fibrillar collagens (cyan) and co-staining for F4/80 (green) to label macrophages. Red shows mTmG graft tissue. Yellow arrowheads indicate macrophages. Each panel shows imaging from a separate individual.

Fusion between nephron epithelia in zebrafish [17] occurs one to two days after epithelial contact, and by analogy, we inferred that fusion events would be evident within the first week after implantation. Kidneys were harvested seven days after engraftment, cryosectioned, and imaged using epifluorescence microscopy with deconvolution. A Texas Red filter set with emission wavelength 630–676 nm was used to register mTmG graft tissue, and a Y5 filter set with emission wavelength 700–776 nm was used to register autofluorescence from the cytoplasm of host epithelial tubules (Figure 1E). At 7 days, the fibrin was resorbed and only organoid tissue could be seen between the capsule and the cortex of the host kidney. mTmG versus autofluorescence yields a detailed view of epithelial tubule morphology at the interface between the graft and the host (Figure 2E,F), and we did not observe any evidence of host:graft tubule fusion in multiple serial sections from the three biological replicates. To confirm the integrity of the graft tubules, we immunostained sections for laminin-1. Interestingly, we found that laminin-1 stains the basement membranes of graft tubules far more intensely than the basement membranes of the adult host (Figure 1G). Basement membranes around epithelial tubules were observed throughout the graft, indicating the tubule structure was retained in the grafted tissue one week after engraftment. Basement membranes were also observed surrounding tubules at the graft:host interface (Figure 1H, arrow). To understand if the barrier between graft and host tubules consisted simply of basement membrane or if there is also significant interstitial collagen representation, we stained cryosections for a panel of interstitial matrix components, including fibronectin, tenascin C, and collagens I, III, V, and VI which were selected based on mass spectrometry analyses of juvenile mouse and adult human kidney cortex [18,19] (Figure 3A–F). 

Focusing our analysis on the interface between the graft and the host, we found that all interstitial matrix components analyzed, with the exception of collagen VI, accumulate between tubules of the graft and host, forming a substantial barrier in which collagens III and V are particularly abundant. Thus, a connection of graft and host epithelia must overcome this physical barrier, which displays similarities to the foreign body response, which is an inflammatory response common to many implantable devices [20]. The NOD.Cg-Prkdcscid Il2rgtm1Wjl/SzJ (NSG) mice used as hosts in these grafting experiments have dysfunctional macrophages and dendritic cells [21], and the only source of functional macrophages is the immunocompetent graft. To determine if macrophages are present in grafts and possibly driving the extracellular matrix accumulation seen at the host:graft interface, we co-stained sections with a cocktail of antibodies for collagens I and III with an antibody that recognizes F4/80, which is present on macrophages (Figure 3G–I). While macrophages can be seen in mTmG grafts in three different individuals, they do not appear to localize specifically at the host:graft interface (Figure 3G–I, yellow arrowheads). Thus, it is possible that macrophages contribute to the extracellular matrix deposition seen in the graft.

We propose to systematically investigate factors that influence matrix barrier formation between graft and host using a screening platform in which organoids are embedded together with candidate recombinant proteins, small molecules, feeder cells, and gene therapy vectors. While most of these factors can either be purchased or generated quite easily in the laboratory, gene therapy vectors that can be used for local gene delivery remain to be identified.

### 3.2. Selection of a Gene Therapy Vector for Local Delivery in Fibrin Hydrogel

**Figure 4:**
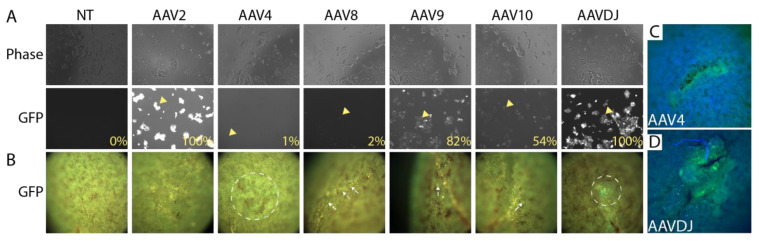
Screening adeno-associated virus (AAV) serotypes for gene delivery at the subcapsular implantation site. (**A**). 293T cells were transduced with a virus expressing EGFP under the control of the CMV promoter/enhancer. Phase images show similar cell densities between samples. Imaging of the GFP channel shows efficient delivery by AAV2, AAV9, and AAVDJ. Arrowheads show examples of transduced cells (**B**). 2 × 10^10^ virus particles of each serotype were embedded in 30 mg/mL fibrin and embedded under the kidney capsule, and the implantation site was visualized by fluorescence stereomicroscopy after 7 days. Despite considerable autofluorescence in the GFP channel, elevated fluorescence could be detected in kidneys engrafted with AAV4 and AAVDJ (dotted circles), while AAV8, AAV9, and AAV10 displayed only sparse transduction of individual cells (arrows). The experiment was performed in biological duplicate, and representative data is shown. (**C**,**D**). Higher magnification images of implantation sites of AAV4 and AAVDJ showing clear GFP signal.

Adeno-associated virus (AAV) provides long-term transduction with limited cytotoxicity and has been used successfully as a gene therapy vector [22]. AAV serotypes are categorized on the basis of receptors that they use to infect cells, and we selected a representative from each group to evaluate local infectivity under the kidney capsule. AAV2 binds heparan sulfate proteoglycan; AAV4 binds sialic acid; AAV9 binds galactose [23]. Previous reports have revealed kidney tropism of AAV8 [24,25], and we also included this serotype. AAV-DJ, which is a chimera of eight different AAVs of human and non-human origin, was also included since it has been shown to transduce effectively in vivo in mice [26]. A panel of these different serotypes carrying CMV-EGFP was tested on HEK293 cells to confirm their infectivity (Figure 4A). All virus preparations tested showed some potential to transduce HEK293 cells, although the frequency of cells transduced by AAV4 and AAV8 was only in the 1–2% range. The capacity of each distinct serotype to transduce locally under the kidney capsule was tested by embedding 2 × 10^10^ copies (determined by QPCR) of each virus in fibrin and implanting. Implantation was performed in biological duplicate. Kidneys were harvested 2 weeks after surgery and evaluated by fluorescent microscopy. Negligible transduction was seen with AAVs 2, 8, 9, and 10, while AAVs 4 and DJ showed evidence of GFP expression by fluorescence stereomicroscopy (Figure 4B). Epifluorescence microscopy confirmed local GFP expression at the sites of AAV4 (Figure 4C) and AAVDJ (Figure 4D) engraftment. Since AAVDJ displayed the strongest GFP signal, we selected this virus for further analysis.

**Figure 5:**
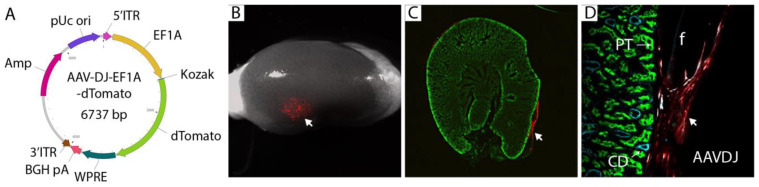
Redesign of AAV gene therapy vector and confirmation of local transduction by engraftment under the kidney capsule. (**A**). Vector design using EF1A promoter and dTomato fluorescent protein. dTomato can be exchanged for genes of interest for in vivo expression at the implantation site. (**B**). 2 × 10^10^ virus particles of AAV-DJ-EF1A-dTomato were embedded in fibrin and implanted under the kidney capsule. Kidneys were harvested after 7 days, and fluorescence (arrow) was visualized by stereomicroscopy. The experiment was conducted in triplicate, and representative images are shown. (**C**). Cryosections of the engrafted host kidney revealed subcapsular gene delivery in the region immediately surrounding the fibrin graft (arrow). (**D**). Cryosections stained with lotus lectin (green) for proximal tubule (PT) and cytokeratin 8 (cyan) for collecting duct (CD) did not show gene delivery into epithelial cells at the graft: host interface.

GFP signal is obscured by strong tissue autofluorescence (Figure 4B), and we chose to use dTomato to confirm the observation from our AAV serotype screen. The CMV promoter is prone to silencing in vivo [27], and we switched to the human elongation factor 1α (EF1A) promoter, which has a similar strength to CMV but displays less variable expression in different cell types [28], suggesting it is less prone to inactivation. The design of the AAV-DJ-EF1A vector that we propose as a vehicle for the delivery and expression of genes to condition the local graft environment is shown in Figure 5A. To test the in vivo potential for local gene expression, we encapsulated 2 × 10^10^ copies of the virus per fibrin clot and engrafted them under the kidney capsule of three immunocompromised mice. After 7 days, local expression at the implantation site could be clearly visualized by low-power stereomicroscopy (Figure 5B, arrow). Kidneys were cryosectioned and stained with markers for the tubules found directly under the capsule: lotus lectin (LTL), which labels the proximal tubule, and cytokeratin 8 which labels collecting ducts. While dTomato expression was intense in cells of fibroblast morphology surrounding the graft (Figure 5C, arrow), we did not observe any evidence of expression in epithelial cells of the proximal tubule or collecting duct (Figure 5D). We also observed that the fibrin clot was not resorbed, unlike implanted clots containing kidney organoids (Figure 2D), indicating that the organoids, rather than the host kidney, degrade the fibrin matrix. From these studies, we conclude that AAV-DJ-EF1A-dTomato gives strong local gene expression at the engraftment site and that expression is restricted to interstitial cells between the capsule and the cortex. Kinetics of vector expression in epithelial cells is likely slower than in interstitial cells, which could be revealed by a longer time course. However, fusion between nephron epithelia in zebrafish [17] and in a mammalian cell culture model (not shown) indicates that this process occurs one to two days after epithelial contact. By analogy, we propose that the critical phase for epithelial fusion between epithelia in kidney organoids and the host kidney is within the first week after implantation, which will be the focus of future screening efforts. For this reason, we limited our gene expression analysis to 7 days.

### 3.3. Identification of Human-Specific Urinary Biomarkers That Can Be Detected in Mouse Urine

**Figure 6:**
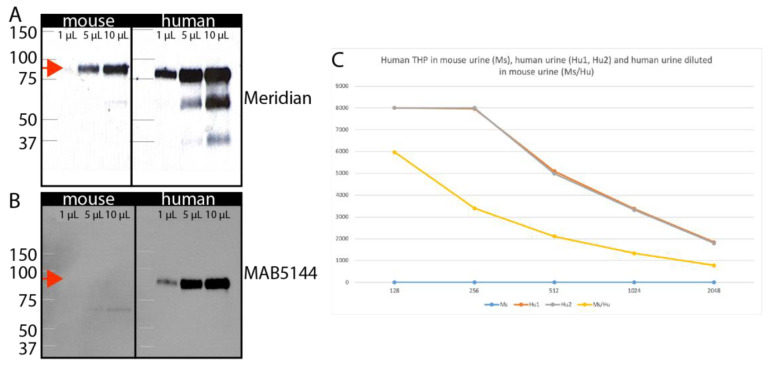
Human-specific uromodulin assay. (**A**). Immunoblot of 1, 5, or 10 µL mouse and human urine with the Meridian sheep anti-uromodulin antibody that detects both species reveal an approximately 90 kD band (arrows). (**B**). Immunoblot of 1, 5, or 10 µL mouse and human urine with the R&D MAB5144 antibody shows a 90 kD band only in human urine. (**C**). ELISA assay using MAB5144 of serial dilutions of human urine (brown), mouse urine (blue), or human urine diluted in mouse urine (yellow).

Visualizing possible tubule fusion events in serial cryosections is a simple and thorough approach to analyzing the graft:host interface (Figure 2E), but proof for functional epithelial tubule fusion requires orthogonal validation. For this purpose, we hypothesized that active secretion of proteins from cells of the nephron into the urine could provide valuable biomarkers that would report on tubule connection. Because the graft is human and the host is a mouse, a species-specific antibody could identify proteins secreted from human nephrons against the background of mouse urine, confirming that graft nephrons are producing filtrate that is passed into the collecting system of the mouse. This would provide strong validation that epithelial tubule connections between the graft and host have been established. To test this concept, we determined if it was feasible to identify commercial reagents that could detect uromodulin (Tamm Horsfall protein) in a species-specific manner. Uromodulin is produced and secreted into the nephron lumen by epithelial cells of the distal nephron [29] and acts as an anti-bacterial agent in urine. The primary sequence is 77% identical between human and mouse at the amino acid level. Using a widely published antibody that has been reported to detect both human and mouse uromodulin [30,31], we confirmed abundant protein in mouse and human urine (Figure 6A). For both species, urine samples from five males and five females were batched for analysis. Several other antibodies against uromodulin are available from commercial vendors, and a comparison between these showed that the MAB5144 antibody from R&D Systems, Minneapolis, MN, USA was a good candidate because (i) the primary sequence used as immunogen includes several stretches of primary sequence that differ between human and mouse, and (ii) it is a mouse monoclonal antibody, most likely detecting a single epitope that differs from mouse. Immunoblot of human versus mouse urine demonstrated that this antibody is specific for human uromodulin and does not detect the mouse protein (Figure 6B). To maximize sensitivity of immunodetection, we used this antibody in an enzyme-linked immunosorbent assay (ELISA) with either mouse urine, human urine, or mixtures of the two (Figure 6C). A two-fold dilution series of human urine starting at 1:128 revealed saturation of the assay at the highest concentration and an approximately linear response down to 1:2048. Quantification versus standard curve revealed that human uromodulin concentration in urine was 32 µg/mL, which corresponds well with published work [32]. Mouse urine showed no signal at any concentration, concurring with the data from immunoblotting (Figure 1B). To determine the effect of mouse urine on the detection of the human protein, we repeated the dilution series of human urine, but instead of using ELISA buffer as the diluent, we used mouse urine. While we do find that the sensitivity of the assay is reduced, we also observe a linear response down to 1:2048, and estimate that the effect of inhibitors in mouse urine would lead to a 2-fold under-estimate of uromodulin concentration. The studies demonstrate that ELISA with MAB5144 provides a simple solution for human-specific nephron biomarker detection in mouse urine.

**Figure 7:**
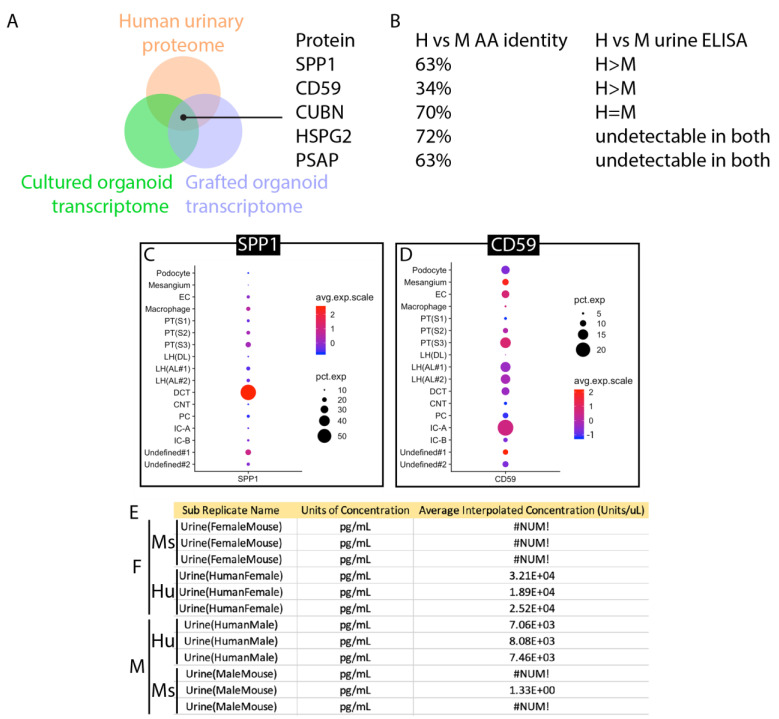
Identification of CD59 as a human-specific biomarker for the proximal nephron. (**A**). Schematic of the screening approach: normal human urinary proteome, in vitro cultured kidney organoid transcriptome, and transcriptome of engrafted kidney organoids were cross-referenced for common proteins/transcripts. (**B**). Amino acid identities of human versus mouse paralogs and results of measurement of human versus mouse using commercial ELISA kits. (**C**). Expression of SPP1 in adult human kidney. (**D**). Expression of CD59 in adult human kidney. (**E**). Results of ELISA measurement of 3 male and 3 female urine samples from humans and mice. #NUM! indicates a measurement below the threshold of detection, 3.21 × 10^4^ is the Glomax plate reader convention for 3.21 × 10^4^. (**F**). Schematic showing the origin of CD59 secretion (green) and uromodulin (THP secretion (purple).

To develop a strategy based on the detection of protein secretion by the proximal nephron, we screened available ‘omic resources to predict candidates. The normal human urinary proteome [33] was used as a basis for analysis and cross-referenced with transcriptomes of human kidney organoids cultured in vitro [34] or engrafted under the kidney capsule [35]. The normal human urinary proteome includes proteins that are secreted into urine by healthy epithelial cells of the nephron, and by intersecting this list with organoid transcriptomes, we aimed to identify naturally secreted biomarkers that are strongly represented in organoid tissue. Each list was ranked by abundance, and the top 50 proteins or genes were compared to identify matches that were represented in all three datasets (Figure S1). The five candidate proteins identified using this approach are shown in Figure 7A. Amino acid identity between human and mouse ranged from only 34% for CD59 to 72% for HSPG2 (Figure 7B), making CD59 the top candidate for a species-specific assay. Commercial ELISA assays were tested using batched human and mouse urine as described for uromodulin. ELISAs for HSPG2 and PSAP did not yield detectable signals using any urine samples, while the assay for CUBN yielded equivalent signals for human and mouse urine. Only ELISA assays for SPP1 and CD59 showed stronger signals in human urine (Figure 7B). Comparison of SPP1 (Figure 7C) versus CD59 (Figure 7D) in human kidneys using the Kidney Interactive Transcriptomics analysis tool [36] demonstrated that CD59 is expressed in cells of the proximal nephron, including both proximal tubule and podocyte. CD59 protein expression has been reported in the human glomerulus and proximal tubule [37,38], where animal models have shown that it protects cells from complement-mediated injury [39]. Reanalysis of human and mouse urine samples from individuals revealed a range of 7.06 to 32.1 ng/mL in human urine and no detectable signal in mouse urine (Figure 7E), confirming that this CD59 ELISA can be used as a human-specific biomarker.

## 4. Discussion

**Figure 8:**
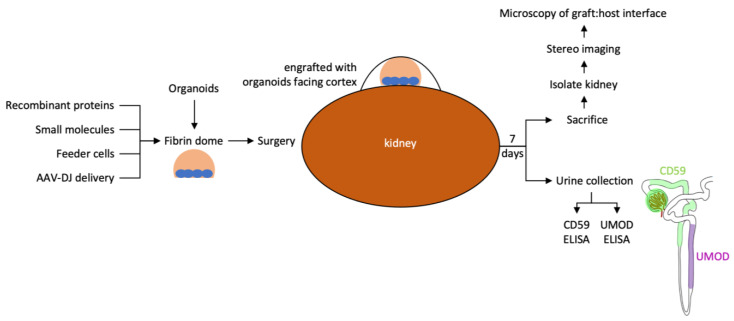
Workflow for testing the effect of local modifiers of the engraftment environment on epithelial connection between graft and host.

This report describes our pipeline to evaluate the epithelial tubule connection between kidney organoid grafts and the host kidney (Figure 8). We have not found examples of processes in the healthy adult human or mouse that connect established epithelial tubules, resulting in the formation of a contiguous lumen. In adult tubular organs such as the kidney, there are significant architectural barriers that separate neighboring tubules, and fusion appears to be a feature that is limited to the fetal period in these species. We anticipate that biological pathways novel to the mouse or human will need to be employed to promote epithelial fusion and provide the conduit for urine output from kidney organoid grafts. Using the testing platform described in this report, we aim to determine if epithelial connection processes can be recapitulated in the adult mouse host to establish a conduit for urine outflow from implanted kidney organoids into the collecting system of the adult mouse host.

Our choice of fibrin as a vehicle to incorporate organoids together with proteins, feeder cells, or viral delivery vectors that can condition the local environment was based on the natural occurrence of clotting at the graft location. This biomaterial is sufficiently rigid so that it can easily be manipulated with forceps, which provides a simple solution for surgical implantation. Interestingly, fibrinolytic processes appear to be very slow in the NSG immunocompromised mouse, and implanted fibrin clots remain largely intact 7 days after implantation. In contrast, when kidney organoids are incorporated into the fibrin clots, the fibrin hydrogel is entirely degraded after 7 days, implying that kidney organoids have strong fibrinolytic activity.

Characterization of the extracellular matrix environment at the interface between the kidney organoid graft and the host kidney demonstrates an important obstacle to epithelial connection: the formation of a collagen barrier. Fibrillar collagens I, III, and V are abundant at the interface, and either suppressing this matrix deposition or degrading it are predicted to be prerequisites to generating an interface in which host and graft tubules directly abut each other. We believe that this is a necessary condition for the fusion of epithelial tubules, and the experimental platform that we are using will allow us to test the potential of metalloproteases and other matrix-degrading enzymes to clear the interstitial matrix barrier between graft and host. Inflammatory cells introduced with immunocompetent grafts are a potential source of extracellular matrix, and anti-inflammatory treatment of the graft is also a candidate strategy to reduce matrix deposition.

An important feature of the strategy to condition the local graft environment is the capacity to incorporate a variety of different delivery vehicles. While recombinant proteins and small molecule pathway modifiers are obvious candidates, their half-lives are short and they diffuse easily. This class of additives would be appropriate to elicit very short-term effects. For extended delivery, feeder cells that produce molecules of interest are an attractive alternative. Feeder cells expressing a variety of factors have been reported, and several are commercially available. One refinement of this approach is the possibility of engineering these cells to produce the factor of interest inducibly, following treatment with doxycycline or tamoxifen, which would enable temporal control. Similarly, AAV has been used extensively as a delivery vector, and virus templates with many different factors are available either commercially or through collaboration. The AAV system is versatile because each template can be used to generate different serotypes to target cells with differing receptor profiles. Understanding which serotype will deliver the payload most efficiently to a cell type of choice largely depends on empirical testing, and our analysis shows that the synthetic DJ serotype provides efficient delivery to the subcapsular graft site. Gene delivery to the interstitial cells in the immediate vicinity of the graft provides a useful strategy to reduce interstitial collagen since these cells are predicted to be an important source of interstitial collagen components [40].

The strategy for human-specific biomarker detection in mouse urine as a read-out of successful epithelial fusion with the formation of a conduit for urine drainage is dependent on the representation of nephron segments that produce the biomarker of interest in kidney organoids. A combination of the CD59 marker for the proximal nephron and the uromodulin marker for the distal nephron is anticipated to detect tubule segments present in the majority of kidney organoids reported to date [5,6,7]. The differentiation protocols for these organoids are similar, and reports have demonstrated successful engraftment with vascularization and formation of filtrate [10,11], which is why we have focused our work on them. More recently, directed differentiation protocols have been reported for reliable differentiation of the collecting duct lineage [41,42]. A human-specific biomarker assay for the collecting duct will need to be developed for the detection of the fusion of these organoids.

Using the platform described in this report, we propose to screen for factors that promote epithelial tubule connection between kidney organoid grafts and adult mouse host kidneys. The development of a tubule fusion strategy will remove a very significant obstacle to the clinical translation of kidney organoid technology for regenerative medicine applications.

## Data Availability

Not applicable.

## Supplementary Materials

The following supporting information can be downloaded at https://www.mdpi.com/article/10.3390/bioengineering10111261/s1, Figure S1: Method and data sources for comparison of healthy human urinary proteome versus human kidney organoid transcriptomes; Table S1: Antibodies and lectins used in the study.

## References

[1] Hoerger T.J., Simpson S.A., Yarnoff B.O., Pavkov M.E., Ríos Burrows N., Saydah S.H., Williams D.E., Zhuo X. (2015). The Future Burden of CKD in the United States: A Simulation Model for the CDC CKD Initiative. Am. J. Kidney Dis..

[2] United States Renal Data System (2021). 2021 USRDS Annual Data Report: Epidemiology of Kidney Disease in the United States.

[3] Kaballo M.A., Canney M., O’Kelly P., Williams Y., O’Seaghdha C.M., Conlon P.J. (2018). A Comparative Analysis of Survival of Patients on Dialysis and after Kidney Transplantation. Clin. Kidney J..

[4] Maung S., Sara A.E., Cohen D., Chapman C., Saggi S., Cukor D. (2017). Sleep Disturbance and Depressive Affect in Patients Treated with Haemodialysis. J. Ren. Care.

[5] Takasato M., Er P.X., Chiu H.S., Maier B., Baillie G.J., Ferguson C., Parton R.G., Wolvetang E.J., Roost M.S., Chuva de Sousa Lopes S.M. (2015). Kidney Organoids from Human iPS Cells Contain Multiple Lineages and Model Human Nephrogenesis. Nature.

[6] Taguchi A., Kaku Y., Ohmori T., Sharmin S., Ogawa M., Sasaki H., Nishinakamura R. (2014). Redefining the in Vivo Origin of Metanephric Nephron Progenitors Enables Generation of Complex Kidney Structures from Pluripotent Stem Cells. Cell Stem Cell.

[7] Morizane R., Lam A.Q., Freedman B.S., Kishi S., Valerius M.T., Bonventre J.V. (2015). Nephron Organoids Derived from Human Pluripotent Stem Cells Model Kidney Development and Injury. Nat. Biotechnol..

[8] Oxburgh L., Carroll T.J., Cleaver O., Gossett D.R., Hoshizaki D.K., Hubbell J.A., Humphreys B.D., Jain S., Jensen J., Kaplan D.L. (2017). (Re)Building a Kidney. J. Am. Soc. Nephrol..

[9] Kobayashi A., Nishinakamura R. (2022). Building Kidney Organoids from Pluripotent Stem Cells. Curr. Opin. Nephrol. Hypertens..

[10] Kumar Gupta A., Sarkar P., Wertheim J.A., Pan X., Carroll T.J., Oxburgh L. (2020). Asynchronous Mixing of Kidney Progenitor Cells Potentiates Nephrogenesis in Organoids. Commun. Biol..

[11] van den Berg C.W., Ritsma L., Avramut M.C., Wiersma L.E., van den Berg B.M., Leuning D.G., Lievers E., Koning M., Vanslambrouck J.M., Koster A.J. (2018). Renal Subcapsular Transplantation of PSC-Derived Kidney Organoids Induces Neo-Vasculogenesis and Significant Glomerular and Tubular Maturation In Vivo. Stem Cell Rep..

[12] Ryan A.R., England A.R., Chaney C.P., Cowdin M.A., Hiltabidle M., Daniel E., Gupta A.K., Oxburgh L., Carroll T.J., Cleaver O. (2021). Vascular Deficiencies in Renal Organoids and Ex Vivo Kidney Organogenesis. Dev. Biol..

[13] van den Berg C.W., Koudijs A., Ritsma L., Rabelink T.J. (2020). In Vivo Assessment of Size-Selective Glomerular Sieving in Transplanted Human Induced Pluripotent Stem Cell-Derived Kidney Organoids. J. Am. Soc. Nephrol..

[14] Brown A.C., Muthukrishnan S.D., Oxburgh L. (2015). A Synthetic Niche for Nephron Progenitor Cells. Dev. Cell.

[15] Shultz L.D., Goodwin N., Ishikawa F., Hosur V., Lyons B.L., Greiner D.L. (2014). Subcapsular Transplantation of Tissue in the Kidney. Cold Spring Harb. Protoc..

[16] van Hinsbergh V.W., Collen A., Koolwijk P. (2001). Role of Fibrin Matrix in Angiogenesis. Ann. N. Y. Acad. Sci..

[17] Kamei C.N., Gallegos T.F., Liu Y., Hukriede N., Drummond I.A. (2019). Wnt Signaling Mediates New Nephron Formation during Zebrafish Kidney Regeneration. Development.

[18] Lipp S.N., Jacobson K.R., Hains D.S., Schwarderer A.L., Calve S. (2021). 3D Mapping Reveals a Complex and Transient Interstitial Matrix During Murine Kidney Development. J. Am. Soc. Nephrol..

[19] Bond K.H., Chiba T., Wynne K.P.H., Vary C.P.H., Sims-Lucas S., Coburn J.M., Oxburgh L. (2021). The Extracellular Matrix Environment of Clear Cell Renal Cell Carcinoma Determines Cancer Associated Fibroblast Growth. Cancers.

[20] Capuani S., Malgir G., Chua C.Y.X., Grattoni A. (2022). Advanced Strategies to Thwart Foreign Body Response to Implantable Devices. Bioeng. Transl. Med..

[21] Shultz L.D., Schweitzer P.A., Christianson S.W., Gott B., Schweitzer I.B., Tennent B., McKenna S., Mobraaten L., Rajan T.V., Greiner D.L. (1995). Multiple Defects in Innate and Adaptive Immunologic Function in NOD/LtSz-Scid Mice. J. Immunol. Baltim. Md. 1950.

[22] Issa S.S., Shaimardanova A.A., Solovyeva V.V., Rizvanov A.A. (2023). Various AAV Serotypes and Their Applications in Gene Therapy: An Overview. Cells.

[23] Srivastava A. (2016). In Vivo Tissue-Tropism of Adeno-Associated Viral Vectors. Curr. Opin. Virol..

[24] Ito K., Chen J., Khodadadian J.J., Vaughan E.D., Lipkowitz M., Poppas D.P., Felsen D. (2008). Adeno-Associated Viral Vector Transduction of Green Fluorescent Protein in Kidney: Effect of Unilateral Ureteric Obstruction. BJU Int..

[25] Chung D.C., Fogelgren B., Park K.M., Heidenberg J., Zuo X., Huang L., Bennett J., Lipschutz J.H. (2011). Adeno-Associated Virus-Mediated Gene Transfer to Renal Tubule Cells via a Retrograde Ureteral Approach. Nephron Extra.

[26] Grimm D., Lee J.S., Wang L., Desai T., Akache B., Storm T.A., Kay M.A. (2008). In Vitro and in Vivo Gene Therapy Vector Evolution via Multispecies Interbreeding and Retargeting of Adeno-Associated Viruses. J. Virol..

[27] Mehta A.K., Majumdar S.S., Alam P., Gulati N., Brahmachari V. (2009). Epigenetic Regulation of Cytomegalovirus Major Immediate-Early Promoter Activity in Transgenic Mice. Gene.

[28] Qin J.Y., Zhang L., Clift K.L., Hulur I., Xiang A.P., Ren B.-Z., Lahn B.T. (2010). Systematic Comparison of Constitutive Promoters and the Doxycycline-Inducible Promoter. PLoS ONE.

[29] LaFavers K., Garimella P.S. (2023). Uromodulin: More than a Marker for Chronic Kidney Disease Progression. Curr. Opin. Nephrol. Hypertens..

[30] Stricklett P.K., Taylor D., Nelson R.D., Kohan D.E. (2003). Thick Ascending Limb-Specific Expression of Cre Recombinase. Am. J. Physiol. Renal Physiol..

[31] Belge H., Gailly P., Schwaller B., Loffing J., Debaix H., Riveira-Munoz E., Beauwens R., Devogelaer J.-P., Hoenderop J.G., Bindels R.J. (2007). Renal Expression of Parvalbumin Is Critical for NaCl Handling and Response to Diuretics. Proc. Natl. Acad. Sci. USA.

[32] Lau W.-H., Leong W.-S., Ismail Z., Gam L.-H. (2008). Qualification and Application of an ELISA for the Determination of Tamm Horsfall Protein (THP) in Human Urine and Its Use for Screening of Kidney Stone Disease. Int. J. Biol. Sci..

[33] Zhao M., Li M., Yang Y., Guo Z., Sun Y., Shao C., Li M., Sun W., Gao Y. (2017). A Comprehensive Analysis and Annotation of Human Normal Urinary Proteome. Sci. Rep..

[34] Phipson B., Er P.X., Combes A.N., Forbes T.A., Howden S.E., Zappia L., Yen H.-J., Lawlor K.T., Hale L.J., Sun J. (2019). Evaluation of Variability in Human Kidney Organoids. Nat. Methods.

[35] Subramanian A., Sidhom E.-H., Emani M., Vernon K., Sahakian N., Zhou Y., Kost-Alimova M., Slyper M., Waldman J., Dionne D. (2019). Single Cell Census of Human Kidney Organoids Shows Reproducibility and Diminished Off-Target Cells after Transplantation. Nat. Commun..

[36] Wu H., Malone A.F., Donnelly E.L., Kirita Y., Uchimura K., Ramakrishnan S.M., Gaut J.P., Humphreys B.D. (2018). Single-Cell Transcriptomics of a Human Kidney Allograft Biopsy Specimen Defines a Diverse Inflammatory Response. J. Am. Soc. Nephrol..

[37] Rooney I.A., Davies A., Griffiths D., Williams J.D., Davies M., Meri S., Lachmann P.J., Morgan B.P. (1991). The Complement-Inhibiting Protein, Protectin (CD59 Antigen), Is Present and Functionally Active on Glomerular Epithelial Cells. Clin. Exp. Immunol..

[38] Ichida S., Yuzawa Y., Okada H., Yoshioka K., Matsuo S. (1994). Localization of the Complement Regulatory Proteins in the Normal Human Kidney. Kidney Int..

[39] Matsuo S., Nishikage H., Yoshida F., Nomura A., Piddlesden S.J., Morgan B.P. (1994). Role of CD59 in Experimental Glomerulonephritis in Rats. Kidney Int..

[40] Ren S., Duffield J.S. (2013). Pericytes in Kidney Fibrosis. Curr. Opin. Nephrol. Hypertens..

[41] Kuraoka S., Tanigawa S., Taguchi A., Hotta A., Nakazato H., Osafune K., Kobayashi A., Nishinakamura R. (2020). PKD1-Dependent Renal Cystogenesis in Human Induced Pluripotent Stem Cell-Derived Ureteric Bud/Collecting Duct Organoids. J. Am. Soc. Nephrol..

[42] Shi M., McCracken K.W., Patel A.B., Zhang W., Ester L., Valerius M.T., Bonventre J.V. (2023). Human Ureteric Bud Organoids Recapitulate Branching Morphogenesis and Differentiate into Functional Collecting Duct Cell Types. Nat. Biotechnol..

