# Supplementary material for: In Vivo Assessment of Laboratory-Grown Kidney Tissue Grafts
Source: Bioengineering (Basel). 2023 Oct 29;10(11):1261. doi: 10.3390/bioengineering10111261 (PMC10669198; doi:10.3390/bioengineering10111261)
Supplement: Supplementary file 1 [file bioengineering-10-01261-s001.zip › bioengineering-2564976-supplementary.pdf]

Table S1: Antibodies and lectins used in this study

Antigen	Vendor	Catalog number	Dilution
Collagen I	Rockland	600-401-103	1:100
Collagen III	Rockland	600-401-105	1:100
Collagen V	SouthernBiotech	1350-01	1:100
Collagen VI	SouthernBiotech	1360-01	1:100
F4/80	Santa Cruz Biotechnology	SC52664	1:100
Fibronectin	Millipore Sigma	F3648	1:100
Tenascin C	Millipore Sigma	T3413	1:100
Laminin	Millipore Sigma	L9393	1:100
Cytokeratin 8	Developmental Studies Hybridoma Bank	TROMA-I	1:100
Uromodulin	Meridian Life Science	K90071C	1:100
Uromodulin	R&D Systems	MAB5144	1:100
Rabbit Ig-Alexa488	ThermoFisher	A-21206	1:200
Goat Ig-Alexa568	ThermoFisher	A-11057	1:200
Rat Ig-Alexa488	ThermoFisher	A-4269	1:200
Sheep Ig-HRP	Jackson Immunochemicals	713-035-003	1:10,000
Mouse Ig-HRP	Biorad	0300-0108P	1:10,000
**LECTIN**			
Lotus lectin-FITC	Vector Laboratories	FL-1321-2	1:200

Figure S1: Method and data sources for comparison of healthy human urinary proteome versus human kidney organoid transcriptomes

Approach to identifying candidates from proteome and transcriptome datasets

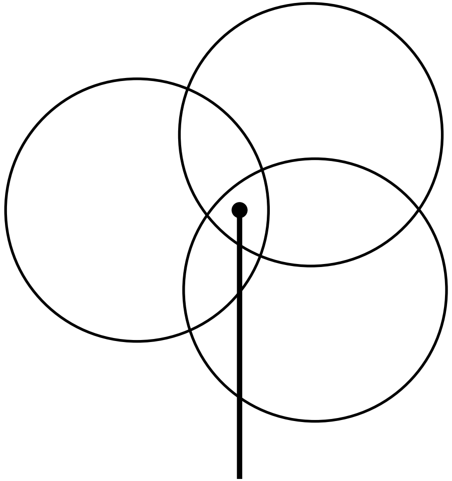

Top 50 hits in each dataset ranked by abundance

Urinary proteome

1	Name	Accesio	Name	MW	iBAQ value	Relative Abunda	L
2	ALB	P02768	Serum albumin OS=Homo sapiens GN=ALB PE=1 SV=2	6.93E+04	4.25E+06	2.94E+11	
3	UMOD	P07911	Uromodulin OS=Homo sapiens GN=UMOD PE=1 SV=1	6.97E+04	4.18E+06	2.91E+11	
4	AMBP	P02760	Protein AMBP OS=Homo sapiens GN=AMBP PE=1 SV=1	3.90E+04	2.70E+06	1.05E+11	
5	IGKC	P01834	Ig kappa chain C region OS=Homo sapiens GN=IGKC PE=1 SV=1	1.16E+04	7.83E+06	9.09E+10	
6	KNG1	P01042	Kininogen-1 OS=Homo sapiens GN=KNG1 PE=1 SV=2	7.19E+04	1.02E+06	7.35E+10	
7	IGHG2	P01859	Ig gamma-2 chain C region OS=Homo sapiens GN=IGHG2 PE=1 SV=2	3.59E+04	1.85E+06	6.63E+10	
8	IGHG1	P01857	Ig gamma-1 chain C region OS=Homo sapiens GN=IGHG1 PE=1 SV=1	3.61E+04	1.60E+06	5.78E+10	
9	APOD	P05090	Apolipoprotein D OS=Homo sapiens GN=APOD PE=1 SV=1	2.13E+04	2.35E+06	5.00E+10	
10	PTGDS	P41222	Prostaglandin-H2 D-isomerase OS=Homo sapiens GN=PTGDS PE=1 SV=1	2.10E+04	2.22E+06	4.68E+10	
11	SPP1	P10451	Osteopontin OS=Homo sapiens GN=SPP1 PE=1 SV=1	3.54E+04	1.29E+06	4.57E+10	
12	CD59	P13987	CD59 glycoprotein OS=Homo sapiens GN=CD59 PE=1 SV=1	1.42E+04	2.96E+06	4.19E+10	
13	IGHG3	P01860	Ig gamma-3 chain C region OS=Homo sapiens GN=IGHG3 PE=1 SV=2	4.13E+04	9.25E+05	3.82E+10	
14	HSPG2	P98160	Basement membrane-specific heparan sulfate proteoglycan core protein O	4.69E+05	7.94E+04	3.72E+10	
15	NDRG2	Q9UN36	Protein NDRG2 OS=Homo sapiens GN=NDRG2 PE=1 SV=2	4.08E+04	9.05E+05	3.69E+10	
16	IGHA1	P01876	Ig alpha-1 chain C region OS=Homo sapiens GN=IGHA1 PE=1 SV=2	3.76E+04	8.44E+05	3.17E+10	
17	EGF	P01133	Pro-epidermal growth factor OS=Homo sapiens GN=EGF PE=1 SV=2	1.34E+05	2.35E+05	3.15E+10	
18	IGHG4	P01861	Ig gamma-4 chain C region OS=Homo sapiens GN=IGHG4 PE=1 SV=1	3.59E+04	8.31E+05	2.98E+10	
19	RNASE2	P10153	Non-secretory ribonuclease OS=Homo sapiens GN=RNASE2 PE=1 SV=2	1.83E+04	1.60E+06	2.93E+10	
20	ITIH4	Q14624	Inter-alpha-trypsin inhibitor heavy chain H4 OS=Homo sapiens GN=ITIH4 P	1.03E+05	2.72E+05	2.81E+10	
21	VASN	Q6EMK4	Vasorin OS=Homo sapiens GN=VASN PE=1 SV=1	7.17E+04	3.85E+05	2.76E+10	
22	PIK3IP1	Q96FE7	Phosphoinositide-3-kinase-interacting protein 1 OS=Homo sapiens GN=PIK	2.82E+04	9.54E+05	2.69E+10	
23	AMY2A	P04746	Pancreatic alpha-amylase OS=Homo sapiens GN=AMY2A PE=1 SV=2	5.77E+04	4.15E+05	2.39E+10	
24	AZGP1	P25311	Zinc-alpha-2-glycoprotein OS=Homo sapiens GN=AZGP1 PE=1 SV=2	3.42E+04	5.98E+05	2.05E+10	
25	ORM1	P02763	Alpha-1-acid glycoprotein 1 OS=Homo sapiens GN=ORM1 PE=1 SV=1	2.35E+04	8.51E+05	2.00E+10	
26	KRT13	P13646	Keratin, type I cytoskeletal 13 OS=Homo sapiens GN=KRT13 PE=1 SV=4	4.96E+04	3.52E+05	1.75E+10	
27	PSAP	P07602	Proactivator polypeptide OS=Homo sapiens GN=PSAP PE=1 SV=2	5.81E+04	2.73E+05	1.58E+10	
28	KLK1	P06870	Kallikrein-1 OS=Homo sapiens GN=KLK1 PE=1 SV=2	2.89E+04	5.33E+05	1.54E+10	
29	SERPINA5	P05154	Plasma serine protease inhibitor OS=Homo sapiens GN=SERPINA5 PE=1 SV=	4.56E+04	3.36E+05	1.53E+10	
30	WFDC2	Q14508	WAP four-disulfide core domain protein 2 OS=Homo sapiens GN=WFDC2 P	1.30E+04	1.18E+06	1.53E+10	
31	TF	P02787	Serotransferrin OS=Homo sapiens GN=TF PE=1 SV=3	7.70E+04	1.88E+05	1.44E+10	
32	SECTM1	Q8WVN6	Secreted and transmembrane protein 1 OS=Homo sapiens GN=SECTM1 PE=	2.70E+04	5.17E+05	1.40E+10	
33	MASP2	O00187	Mannan-binding lectin serine protease 2 OS=Homo sapiens GN=MASP2 PE=	7.57E+04	1.77E+05	1.34E+10	
34	CD44	P16070	CD44 antigen OS=Homo sapiens GN=CD44 PE=1 SV=3	8.15E+04	1.64E+05	1.34E+10	
35	SERPING1	P05155	Plasma protease C1 inhibitor OS=Homo sapiens GN=SERPING1 PE=1 SV=2	5.51E+04	2.42E+05	1.34E+10	
36	CUBN	O60494	Cubilin OS=Homo sapiens GN=CUBN PE=1 SV=5	3.98E+05	3.27E+04	1.30E+10	
37	LMAN2	Q12907	Vesicular integral-membrane protein VIP36 OS=Homo sapiens GN=LMAN2	4.02E+04	3.20E+05	1.29E+10	
38	CLU	P10909	Clusterin OS=Homo sapiens GN=CLU PE=1 SV=1	5.25E+04	2.38E+05	1.25E+10	
39	KRT4	P19013	Keratin, type II cytoskeletal 4 OS=Homo sapiens GN=KRT4 PE=1 SV=4	5.72E+04	2.16E+05	1.23E+10	
40	RNASE1	P07998	Ribonuclease pancreatic OS=Homo sapiens GN=RNASE1 PE=1 SV=4	1.76E+04	6.99E+05	1.23E+10	
41	AHSG	P02765	Alpha-2-HS-glycoprotein OS=Homo sapiens GN=AHSG PE=1 SV=1	3.93E+04	3.00E+05	1.18E+10	
42	PIGR	P01833	Polymeric immunoglobulin receptor OS=Homo sapiens GN=PIGR PE=1 SV=	8.32E+04	1.39E+05	1.16E+10	
43	GAA	P10253	Lysosomal alpha-glucosidase OS=Homo sapiens GN=GAA PE=1 SV=4	1.05E+05	9.63E+04	1.01E+10	
44	F2	P00734	Prothrombin OS=Homo sapiens GN=F2 PE=1 SV=2	7.00E+04	1.44E+05	1.01E+10	
45	GSN	P06396	Gelsolin OS=Homo sapiens GN=GSN PE=1 SV=1	8.56E+04	1.18E+05	1.01E+10	
46	PGA3	P0DJD8	Pepsin A-3 OS=Homo sapiens GN=PGA3 PE=1 SV=1	4.19E+04	2.24E+05	9.40E+09	
47	KRT1	P04264	Keratin, type II cytoskeletal 1 OS=Homo sapiens GN=KRT1 PE=1 SV=6	6.60E+04	1.38E+05	9.12E+09	
48	LGALS3BP	Q08380	Galectin-3-binding protein OS=Homo sapiens GN=LGALS3BP PE=1 SV=1	6.53E+04	1.37E+05	8.97E+09	
49	RPS12	P25398	40S ribosomal protein S12 OS=Homo sapiens GN=RPS12 PE=1 SV=3	1.45E+04	6.17E+05	8.95E+09	
50	YIPF3	Q9GZM5	Protein YIPF3 OS=Homo sapiens GN=YIPF3 PE=1 SV=1	3.82E+04	2.31E+05	8.82E+09	

Red text = hits represented in cultured organoid and implanted organoid transcriptomes

Candidates selected for further analysis

Spp1 = osteopontin

CD59 = MAC inhibitory protein

HSPG2 = perlecan

PSAP = prosaposin

CUBN = cubilin
